# RADIOPROTECTION CAMPAIGN AND CARD: EDUCATIONAL STRATEGIES THAT REDUCE CHILDREN’S EXCESSIVE EXPOSURE TO RADIOLOGICAL EXAMS

**DOI:** 10.1590/1984-0462/;2017;35;2;00011

**Published:** 2017

**Authors:** Mônica Oliveira Bernardo, Fernando Antonio de Almeida, Flavio Morgado

**Affiliations:** a Pontifícia Universidade Católica de São Paulo, Campus Sorocaba, Sorocaba, SP, Brasil.

**Keywords:** radioprotection, children, health education

## Abstract

**Objectives:**

To analyze the results of an initiative aimed at improving the reasonable use of radiological examinations, ensuring their technical quality, implementing a radioprotection campaign that includes training of the professional team, and introducing the radioprotection card for children under 12 years old as a tool for parents and doctors to control children’s exposure to radiation.

**Methods:**

The study was held in a health care insurance system covering 140,000 people. A radioprotection campaign was implemented according to *Image Gently* ^•^ protocols, ensuring the lowest dose of radiation and the quality of examinations, and the radioprotection card was implemented. To assess the effectiveness of these actions, the number of radiological examinations performed at the pediatric emergency room in a period of one year preceding the campaign was compared with the number of radiological examinations performed one year after the campaign.

**Results:**

The campaign was well accepted by all professionals, families, and patients involved. In the year following the implementation of radioprotection strategies, there was a 22% reduction of radiological examinations performed at the pediatric emergency room. There was also a 29% reduction in the request of two or more radiological examinations for the same child or examinations with two or more incidences.

**Conclusions:**

The campaign and the radioprotection card for children under 12 years old proved to be feasible strategies and correlated with a reduction in radiological examinations requested and performed at the pediatric emergency room.

## RESUMO

**Objetivos:**

Analisar resultados de iniciativa para racionalizar o uso de exames radiológicos, garantindo sua qualidade técnica, e implantar uma campanha de radioproteção que inclua a capacitação da equipe profissional e a Carteira de Radioproteção para crianças de até 12 anos como instrumento para que os pais e médicos controlem a exposição das crianças à radiação.

**Métodos:**

Em um sistema de saúde suplementar com cobertura de 140 mil pessoas, foi implantada a campanha de radioproteção com base nos protocolos *Image Gently* ^•^, garantindo a menor dose possível de exposição à radiação, com qualidade técnica, e implantando a Carteira de Radioproteção. Para aferir a eficácia dessas ações, comparou-se o número de exames radiológicos realizados no setor de urgência e emergência pediátrica no período de um ano anterior à campanha com o número de exames radiológicos realizados no período de um ano posterior à campanha.

**Resultados:**

As ações foram bem-aceitas por todos os grupos envolvidos. No ano seguinte à implantação das estratégias de radioproteção, observou-se redução de 22% no número de exames radiológicos realizados no setor pediátrico de urgência e emergência. Houve ainda diminuição de 29% da solicitação de dois ou mais exames para a mesma criança ou de exames com duas ou mais incidências.

**Conclusões:**

A campanha de radioproteção e a implantação da Carteira de Radioproteção para crianças até 12 anos revelaram-se estratégias factíveis e associaram-se à redução dos exames radiológicos solicitados e realizados no setor de urgência e emergência pediátrica.

## INTRODUCTION

In recent decades, technological advances and the ease of performing radiological examinations, particularly computed tomography (CT), have led to a progressive increase in the request and execution of these examinations.^1^ Although radiological examinations can aid in diagnosis and therefore offer comfort and security for physicians and parents, its excess use has caused concern in the medical and scientific field because of the cumulative effect of ionizing radiation. In the United States, it was estimated that the number of CT scans performed per year was 3 million in the 1980s, increasing to 62 million in 2007.^1^ Where before CT represented approximately 2 or 3% of all radiological examinations, today the rate has risen to 20%-30% due to its wide application and the ease and availability of equipment.^2^ In the UK, CT represents 50% of the radiation dose received in all radiological examinations.^3^

Because their tissues are still in development, children and adolescents are more sensitive and more vulnerable to the effects of ionizing radiation. The younger the patients, the greater the risk.^4^ Several studies indicate an increase in the risk of ionizing radiation exposure in children exposed to CT when compared to non-exposed children. An American study estimated a 0.18% increase in the risk of cancer death in a 1-year-old child after being exposed to radiation from a CT scan of the abdomen, or 0.07% after exposure to a CT scan of the skull, far from being an insignificant risk.^5^ The same study estimates that in the United States at the end of the 1990s, 600,000 CTs of the abdomen or skull were performed per year in children under the age of 15 years. The authors infer that, roughly speaking, 500 of these children would have died of cancer attributed to the examination.^5^ In the United Kingdom, 180,000 children undergoing low-dose tomography were followed-up from 1985 to 2002, and the results indicate an increased incidence of leukemia and brain tumor.^6^ In Australia, a study that tracked 11 million children and adolescents found that those who had undergone CT examination exhibited 24% more risk of developing cancer than the non-radiation exposed population. This risk positively correlated with younger age and number of examinations performed.^4^

In the hospital, in which we conducted this study, the follow-up indicates excessive request for radiological examinations. It is believed that this excess is due to the insecurity of parents and guardians or the health team itself. Although there is often a specific reason for requesting radiological examinations - as they are certainly helpful in making a diagnosis - the fact is that children are at risk of being exposed. This is especially true in the pediatric emergency sector, with large number of visits and requests for radiological examinations.

The objective of this study was to evaluate the initiative of a private hospital (with supplementary health system coverage) aimed at implementing measures to reduce children’s exposure to the risk of ionizing radiation. This initiative likewise aimed at enhancing training and awareness of health professionals and family members of the dangers of excessive radiation. In this initiative, a radioprotection campaign was launched that involved the awareness of employees and those responsible for children; the technical adequacy of radiological equipment according to the radioprotection criteria of the American College of Radiology and the American Society of Pediatrics, and the implementation of the radioprotection card for children up to 12 years of age.^7^ ^,^ ^8^ ^,^ ^9^

## METHOD

This is a quantitative and qualitative prospective study. We evaluated the medical attendance at the emergency room and the proportion of radiological examinations requested, and analyzed the impact on the health service and the clientele served. The study was conducted in a hospital of a supplementary health system run by a medical cooperative in the interior of the state of São Paulo. Its coverage is approximately 140,000 people. Urgent care, emergencies, and subsidiary examinations of the entire health system are centralized in the hospital itself.

The protocols for performing all the radiological examinations were reviewed by the radiologists of the sector, adopting in the execution of these examinations the lowest milliamperage (mA - maximum 100-150 mA) in order to maintain the necessary quality for the examination report.^7^ ^,^ ^8^ The lowest kilovoltage (KV) was used and determined by the 64-channel Philips multislice CT equipment at the time of CT examination. The extent of the field of exposure was limited to the required area. The examination was only initiated after being sure that the positioning of the patient was correct in order to avoid error and repetition of the examination. Daily monitoring ensured that the measures taken in the execution of the examination maintained minimal radiation dose.

As for the health professionals, meetings were held with the management team and the nursing staff so that the employees understood the effects of the radiation, ensuring that they were aware that the radiological examinations should only be done when necessary. Since then, the entire care team (not just the doctor) has explained to family members the risks of radiological examinations, especially of CT. This is important considering that children’s caregivers often believe that a radiological examination is indispensable to diagnosis, rather than the child clinical examination. A medical event was held in which a professor specializing in pediatric radiology addressed the technical aspects and risks of ionizing radiation of radiographs and CT. Information leaflets were distributed to pediatricians about the additive effect of ionizing radiation, the estimated dose of radiation absorbed by exposure to the most requested examinations, and the importance of performing these examinations only when truly necessary. These initiatives engaged health care professionals in the worldwide Image Gently^•^ campaign.^10^

Information regarding the importance of this campaign, the radioprotection card and the reasonable use of radiological examinations was posted in the hospital’s web page and advertised throughout the institution in spaces accessible to both health care professionals and the public. Twenty thousand radioprotection cards were prepared and distributed, providing a tool for patients and health care professionals to keep a record of the type of radiological examination and its date. The back cover provides information in language appropriate for laypeople that explains the need and benefits of the initiative, such as the risk of serious diseases and cancer. The delivery of the radioprotection card was always accompanied by an explanation by a trained employee in the emergency room, examination collection laboratories, hospital imaging sector, and pediatric offices.

In order to evaluate the impact of the awareness campaign and the radioprotection card, radiological examinations and emergency pediatric consultations of this supplementary health system were quantified. These consultations are carried out in one single location and the complementary examinations are mandatorily performed at the hospital and registered on the computerized service card. Thus the record systems allowed for the documentation of the number of radiological examinations (X-ray and CT) associated with all the pediatric consultations performed in the sector. As the launch of the radioprotection campaign took place at the end of August 2013, the previous period from September 2012 to August 2013 was considered as the pre-campaign period for the collection of such data, and the period of one year thereafter - September 2013 to August 2014 - as the post-intervention or post-campaign period.

The study was approved by the Research Ethics Committee of the Faculty of Medical Sciences and Health of the Pontifical Catholic University of São Paulo (PUC-SP). All the procedures of the study were carried out in accordance with the applicable ethical precepts. The review of protocols of examinations was made by consensus by radiologists in the field, and the campaign followed the guidelines of the American College of Radiology and the American Society of Pediatrics.^10^

The data are presented descriptively, and the comparison test between two proportions was used to compare the percentages of attendance with radiological examinations in pre- and post-radioprotection campaign. In order to assess the proportion of visits associated with two or more radiological examinations for the same child, or two or more incidences in the same examination request, a random sample of 1,051 requested examinations from September 2012 to August 2013 (pre-campaign) and 920 requested examinations from September 2013 to August 2014 (post-campaign) were used. To do this, we selected all attendances at pediatric emergency room in the second week of January and June of each year (pre-campaign and post-campaign). The months of January and June were chosen because they were, respectively, the ones with the lowest and highest number of monthly visits in the sector. Samples were selected in the same periods of the previous year and after the radioprotection campaign. The minimum size of these samples was estimated in 640 visits with requests for examinations in each period, so that a minimum difference of 5% could be found between two proportions, taking into account the error α of 5% and the error β of 20%.

## RESULTS

At the launch and in the month following the campaign, approximately 17,000 radioprotection cards were distributed, always accompanied by the necessary clarifications and guidance to its use like a vaccination card, that is, has to be presented whenever medical care was sought. The initiative had significant repercussions in the local media, which contributed to its dissemination.

Pediatricians who initially hesitated to distribute the radioprotection card out of fear that it might bring excessive concern to their parents were surprised by the positive reception of the campaign. They reported that the radioprotection card had become a very useful tool to raise awareness among parents, gaining their confidence and collaboration in order to avoid exposing their children to unnecessary examinations that could endanger their health. The medical coordinator of the pediatric emergency unit expressed her agreement:

*Many parents already arrive at the clinic requesting and pressuring the doctor to request radiological examinations, with no idea of the risk exposed to the children. The preparation of information at the pre-consultation reception and in the doctor’s office helps pediatricians to have scientific tools to raise family awareness. We consider the project of great importance in the safety of medical practice, with improvement in training and prevention of future diseases in children.*

The health system board also showed enthusiasm for the initiative and the results of the radioprotection campaign. The hospital superintendent asserts:

*I believe that this project overcomes the main challenge of any scientific research: to cross the walls of the academy and enter the reality of people and the community, transforming habits through the proposition of feasible solutions [...]. The project of radiological protection in childhood, which, in addition to seeking to perform the tests with the lowest radiation dose possible, also aims to raise awareness of the risks of diagnostic methods that use the X-ray to obtain the images, changing how the care team and clients use these methods.*

**Figure 1:**
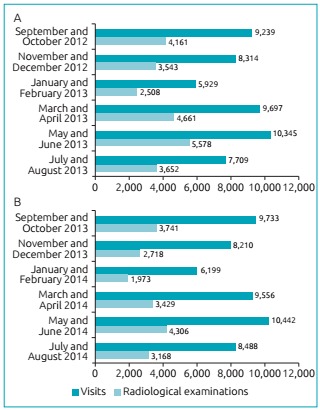
Number of visits and radiological examinations performed in the pediatric emergency sector of a supplementary health system in the interior of São Paulo State: (A) before the Radioprotection Campaign for children up to 12 years of age; (B) after the Radioprotection Campaign for children up to 12 years old.

The rationalization of the radiological examinations was evidenced by comparing the number of examinations made in the pediatric emergency department of the hospital one year before the campaign with the number of examinations one year after the campaign. Figure 1A shows the bi-monthly distribution of the number of visits and the number of radiological examinations performed between September 2012 and August 2013, pre-campaign period. In that year, 51,233 visits and 24,103 radiological examinations occurred, that is, 47% of the visits generated radiological examinations. In the following year, after the radioprotection campaign (Figure 1B), there were 52,628 visits, slightly higher than the previous year, and the number of radiological examinations in the period fell to 19,335, corresponding to 36.7% of the visits. Therefore, considering the number of visits in each period, there was a reduction of 22% in the execution of radiological examinations after the campaign and the distribution of radioprotection cards. This reduction was consistent throughout the follow-up period of one year. The difference between the proportions of radiological examinations performed in each period is statistically significant (p <0.001).

**Figure 2:**
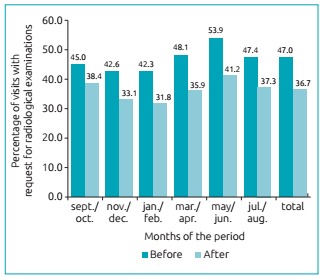
Percentage of visits that generated radiological examinations performed in the pediatric emergency sector of a supplementary health system in the interior of São Paulo State before and after the Radioprotection Campaign for children up to 12 years old.

Figure 2 shows the percentage distribution of pediatric emergency room visits with radiological examinations before and after the campaign and the implementation of the radioprotection card. As observed, regardless of the period analyzed, the number of radiological examinations was reduced after the Radioprotection Campaign, and the difference remained throughout the observed period.

Table 1 shows the types of radiological examinations most performed in children in the emergency sector. As noted, there is extensive primacy of examinations to evaluate the respiratory system. Chest x-rays and facial sinuses are the most requested and often both are indicated for the same child. Among CT scans, CT scans of the skull and abdomen are the most requested (Table 1).

**Table 1:**
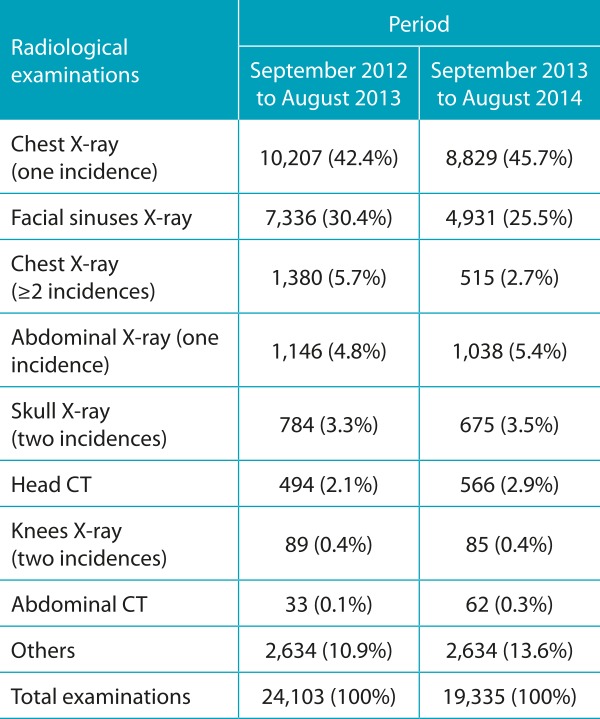
Most frequently requested radiological examinations in the pediatric emergency sector according to the period evaluated.

CT: computed tomography.

Table 2 shows the distribution of two samples of radiological examinations requested in the pediatric emergency and emergency sector in similar periods before and after the Radioprotection Campaign. When analyzing the requests of two or more examinations for the same child, or the examination of a single region with two or more incidences, it is observed that the proportion of request for examinations in duplicate or with two or more incidences was significantly lower in the year post-campaign (p <0.001). Thus, in addition to the reduction in the proportion of consultations in which radiological examinations were requested, there were also decrease in the examinations with two or more incidences or examinations in more than one body region.

**Table 2:**
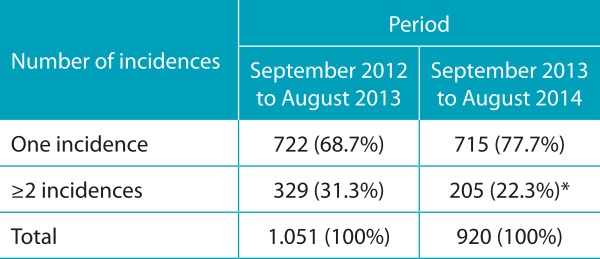
Distribution of the number of incidences of requested radiological examinations in samples randomly selected in similar periods before and after the radioprotection campaign (August 2013).

*p<0.001 to compare two proportions (Z test).

## DISCUSSION

The study model encouraged health professionals to modify their work environment by creating tools to raise the awareness of these professionals and their families about the effects of ionizing radiation on children, therefore establishing a true preventive and health promotion project. Also as a consequence of the project, the radioprotection card was developed and distributed to children up to 12 years old, allowing pediatricians and other physicians to analyze possible previous examinations and decide whether or not to recommend new radiological examinations, reducing radiation exposure. From the qualitative point of view, the improvement of information and knowledge of employees, physicians and users - and particularly of the parents - reflects an important aspect of health education, whose corollary is the promotion of health, a responsibility inherent to professionals in the area. Comprehensive health care in childhood and adolescence reserves a central role for parents, but also for health institutions and their multiprofessional team.^11^

The systematic and sustained reduction (by 22%) of the radiological examinations associated to pediatric care in the emergency sector also allows quantification of the results of the campaign and the implementation of the radioprotection card. At the same time, there was a reduction in the request for duplicate examinations for the same child and examinations with two or more incidences. The study identified the high proportion of pediatric visits associated with radiological examinations in emergency sector, most of them related to respiratory tract diseases, in which clinical evaluation should be given greater weight. In addition, in this setting of care, it is common to request examinations of more than one anatomical region or examinations with two or more incidences. A Brazilian study assessed the care with radiological protection in four large hospitals in children submitted to X-ray of the facial sinuses. It was observed that the protection was incipient and that two incidences were frequently requested unnecessarily.^12^

The project was inspired by the Image Gently^•^ initiative, conducted by the American College of Radiology and the American Society of Pediatrics, and then subsequently carried out in many medical and health entities across the United States.^10^ Here it was adapted to the Brazilian regional reality. The quality sector of the hospital was also involved in the radiation protection project and, in a recent external audit, received praises for the initiative, which auditors believe to be a pioneer in Brazil. We do not know about similar projects in Brazil, but the communication of these results in national and international congresses and the dissemination in other supplementary health systems have aroused great community interest, and similar projects are being developed.^13^

There is consensus in the literature regarding the risks of exposure to ionizing radiation and the development of solid tumors or hematopoietic lineage in children and adolescents.^1^ ^,^ ^3^ ^,^ ^6^ However, some authors in France have drawn attention to the possibility that the design of these studies determine a reverse causality. That is, the children selected in the studies are exposed to the radiological examinations because they already presented some abnormality and, therefore, are more predisposed to develop oncological diseases.^14^ To solve this question, a study is underway in which this aspect is “excluded”: the research is carried out with children and adolescents who undergo radiological procedures because they present cardiac problems and who, after the procedures, are followed up to assess the risk of developing cancer.^15^ Until this methodological aspect is clarified, professionals should continue to educate health care providers, parents, and children themselves, guaranteeing them the option of promoting health through radiological protection.

It is worth considering as a limitation of the study the fact that parents or guardians were not interviewed to determine their perception of the campaign and the radioprotection card. In addition, the risk of underdiagnosis of some diseases because of the reduction in the number of radiological examinations was not assessed. Despite these limitations, this study presents the unprecedented experience in Brazil of a proposal for radiological protection for children and adolescents through a Radioprotection Campaign, emphasizing the awareness of health professionals and their families about the possible harm of cumulative ionizing radiation and offering a motivating instrument for the control of radiation exposure - the radioprotection card. The monitoring of radiation exposure in children in a controlled sector of this health system evidenced the consistent reduction of the radiological examinations associated to pediatric emergency care. The enthusiasm with which the project was accepted in this institution and the commitment observed in its execution have ensured its success and must guarantee its permanence.
